# What my body looks like and what my body can do: A self-perception explanation of excessive exercise in young adults with anorexia and/or bulimia

**DOI:** 10.3389/fpsyg.2022.916294

**Published:** 2022-10-17

**Authors:** Marilou Ouellet, Johana Monthuy-Blanc, Robert Pauzé, Michel Rousseau, Stéphane Bouchard

**Affiliations:** ^1^GR2TCA-Loricorps-Groupe de Recherche Transdisciplinaire des Troubles du Comportement Alimentaire, Université du Québec à Trois-Rivières, Trois-Rivières, QC, Canada; ^2^Research Center, Institut Paul Bocuse, Écully, France; ^3^Centre de Recherche de l’Institut Universitaire en Santé Mentale de Montréal, Montréal, QC, Canada; ^4^Department of Psychoeducation, Education Faculty, Université de Sherbrooke, Sherbrooke, QC, Canada; ^5^Department of Psychoeducation, Université du Québec à Trois-Rivières, Trois-Rivières, QC, Canada; ^6^Chaire de Recherche du Canada en Cyberpsychologie Clinique, Université du Québec en Outaouais, Gatineau, QC, Canada

**Keywords:** physical self-concept, body image, virtual reality, physical activity, compulsive exercise

## Abstract

The present study examined the relationships between quantitative and qualitative dimensions of excessive exercise (EE) with the physical self-perception’s dimensions and perceptual perspectives of body image (i.e., allocentric/3rd person and egocentric/1st person perspectives). The *e*LoriCorps Immersive Body Rating Scale 1.1, the very short form version of the Physical Self-Inventory and the Exercise and Eating Disorder test were used. The study includes 36 people with anorexia and/or bulimia seeking an external and specialized transdisciplinary program for eating disorders. Results show a different correlational profile of physical self-perceptions depending on the dimension (qualitative or quantitative) used to define EE. Differences in the perspectives used to assess body dissatisfaction (allocentric or egocentric) were also found. Perceived physical appearance was the key predictor of the qualitative dimension of excessive exercise. Findings suggest that EE in patients with an eating disorder could be explained by the feeling of competence not only related to physical appearance but also to physical abilities.

## Introduction

Eating disorders (ED), and more particularly anorexia nervosa (AN) and bulimia nervosa (BN), are characterized by a disturbance in the perception of body image and self-esteem that implies food intake, weight loss and shape concerns (American Psychological Association, 2013). These disturbances of self-perceptions are related to clinical behaviors. Among these behaviors, excessive exercise (EE, sometimes referred to as Excessive Physical Exercise) is recognized as a common feature of patients with AN and/or BN (Davis et al., 1997; Rizk et al., 2015). Specifically, the EE affects 44–80% of restrictive subtype patients with AN, 43–53% of binge/purging subtype patients with AN and 21–39% of patients with BN (Shroff et al., 2006; Dalle Grave et al., 2008).

Even though physical activity and exercise are related to global well-being in the general population (Hausenblas and Fallon, 2006; Martin et al., 2016), its effects can become particularly deleterious in ED (Hausenblas et al., 2008). Indeed, patients with ED who engage in EE show more severe psychopathology, longer duration of treatment, hospitalizations characterized by multiple readmissions, lower Body Mass Index [BMI (kg/m^2^)] than those who do not, and are more likely to relapse (Solenberger, 2001; Steinhausen et al., 2008; Bratland-Sanda et al., 2010; Vall and Wade, 2017; Young et al., 2018). Furthermore, suicide is the leading cause of death in patients with ED (Fichter and Quadflieg, 2016), and the presence of EE is a significant predictor of suicide attempts (Smith et al., 2013).

As early as 1972, Feighner et al. (1972) recognized some form of hyperactivity in patients with AN. Over time, this observation was followed by a plethora of definitions and differences in prevalence rates and conceptualizations of EE (Davis et al., 1997; Rizk et al., 2015). EE is most often determined by its quantitative dimensions, namely the frequency and duration of exercise done to control body weight and shape (Davis and Fox, 1993). As a reference, several authors reported using the convention initiated by Davis et al.’s (Davis et al., 1997) stating that six or more hours per week of exercise performed for reasons related to body control is sufficient to categorize patients as exercisers, or engaged in EE (Solenberger, 2001). In contrast, several authors highlighted the importance of what they called qualitative dimension of exercises in EE, such as attitudes and thoughts about the compulsivity of exercising, and documented their contribution to the pathological nature of EE (Adkins and Keel, 2005; Boyd et al., 2007; Cook and Hausenblas, 2008; Noetel et al., 2017). Qualitative dimensions of EE involves: (a) unawareness of bodily signals of pain and fatigue, (b) rigid maintenance of the schedule for exercise, (c) prioritization of this exercise at the expense of other daily occupations important to the individual, and finally, (d) expression of feeling distress, shame, and anxiety in the event that exercise is impossible (Taranis et al., 2011; Danielsen et al., 2018).

The relationship between the dimensions of EE and ED is complex (Rizk et al., 2015). A significant challenge faced by researchers is the lack of global consensus on the definition of EE. Indeed, when comparing different subpopulations of adults who exercise excessively, Rizk et al.’s (2015) found that the ED symptomatology was associated, and varied, with how EE was defined and operationalized. Some authors highlighted the synchronous positive and negative reinforcement of exercise in patients with AN, which can produce both pleasure and allow to avoid/neutralize internal distress (Noetel et al., 2017; Watson et al., 2019). Self-reported motives for exercise are multiple (e.g., weight control, regulation of emotional states, giving oneself permission to eat, feeling light, taking a moment for oneself or to clear one’s mind, feeling good during/after exercise, etc.) and change over time (Johnston et al., 2011; Brunet et al., 2021).

EE can play many functions in the development and as maintaining factors of ED. Concerning their affective role, some authors have highlighted that EE can act as an emotional regulation strategy for negative affect related to stress, anxiety and depression (Vansteelandt et al., 2007; Brunet et al., 2021). Concerning their behavioral role, EE has been identified as an inappropriate compensatory behavior that serve to avoid a weight gain by compensating for calories ingested as a result of binge eating episodes (Fairburn et al., 2003; Mond and Calogero, 2009; Bratland-Sanda et al., 2010). This behavior that also contribute to weight lost regardless of the amount of food ingested (Davies, 2015). Research on EE has contributed to refine our understanding of ED and its challenging treatment (Davis and Carter, 2009), yet there is still more to be done.

Exercise is known to influence individual’s global self-esteem and physical self-appearance (Kolnes and Rodriguez-Morales, 2016). Efforts by people with ED to control food intake and body weight and shape are mainly associated with distorted self-perception, or inaccurate perception of how they look, which constitute the core of ED (Cash and Smolak, 2011). Distorted physical self-perceptions refer to the cognitive and affective dimensions of body image disturbances such as body distortion and body dissatisfaction, as well as other perceptual dimensions of body image disturbances such as physical self-worth and physical appearance. Although global self-esteem and relations with the body are central to ED, most of the studies focus on perceived physical appearance and body image, neglecting to include perceived physical abilities. In this regard, it is possible that patients with ED with EE valued themselves through two main different perceptual sources of physical identity; one related to the body image (“what my body looks like?”) and one related to the physical abilities (“what my body can do?”). The model proposed by Fox and Corbin (Fox and Corbin, 1989) notably highlighted that, as part of global self-esteem and physical self-perceptions, perceived physical appearance (i.e., the perception to be able to maintain an attractive body over time) represents only one dimension, which must be complemented by perceived physical abilities such as perceived sport competence, physical condition and physical strength. The study of perceived physical abilities has been neglected in people with ED and should be documented in addition to body image.

Body image distortion is often measured as the discrepancy between estimating one’s own body size and one’s real body size, whereas body image dissatisfaction refers to the discrepancy between estimating one’s own body size and one’s ideal body size (Cash and Smolak, 2011). Studies that focused on the relationship between EE and body image used classical paper questionnaires (e.g., Body scale questionnaire, Rousseau et al., 2005). However, body image is a multidimensional phenomenon that implies affective, cognitive and perceptual dimensions (Monthuy-Blanc et al., 2020, 2022). That complexity needs to be assessed through measures that allow to reflect those dimensions. In recent years, immersions in virtual reality (VR) has been used to measure body-image distortion and body-image dissatisfaction, the two main components of distorted physical self-perception (Riva and Melis, 1997; Fisher et al., 2020). VR has the advantage of providing information from two different perspectives: (a) allocentric (i.e., the third-person perspective of reference, with the body being perceived as an object observed integrated into the surrounding physical environment); and (b) egocentric (i.e., the first-person perspective of reference, as perceived and experienced through one’s eyes; Corno et al., 2018; Riva, 2018; Fisher et al., 2020; Monthuy-Blanc et al., 2020). The egocentric perspective allows the feeling of embodiment and offers new insight on how the body is experienced and perceived based on intra-individual comparison, particularly for body distortion, rather than inter-individual comparison. The later referring to the allocentric perspective and the affective-attitudinal dimension of body image often measured with figure rating scales, for example (Monthuy-Blanc et al., 2020).

In sum, three research domains in the field of ED are important and their mutual relationship has never been investigated: (a) the quantitative and qualitative dimensions of EE, (b) physical self-perception of both body image and physical abilities, and (c) body image investigated from the allocentric as well as the egocentric perceptual perspectives. The objective of this correlational study conducted with people seeking treatment for ED was to document the associations between dimensions of EE, dimensions of the physical-perceptions self, and perceptual dimensions of body image. The analyses focused on three research questions: Q1 – what are the relations between EE and physical self-perception, and do they differ when looking at qualitative versus quantitative dimensions of EE; Q2 – what are the relations between EE and body image, and do they differ when looking at allocentric versus egocentric perspectives, Q3 – what are the relations between perceived physical self and body image, and to they differ when looking at allocentric versus egocentric perspectives?

## Materials and methods

### Participant and procedure

The initial sample consisted of people seeking treatment for eating disorders in a transdisciplinary-university-based program named *e*LoriCorps^1^ from September 2016 to June 2019. AN and BN diagnoses were established by clinicians and the clinical transdisciplinary team from the Transdisciplinary research group on eating disorder, now called – Loricorps – of UQTR (Monthuy-Blanc et al., 2016) respecting DSM-5 criteria. The inclusion criteria were to: (a) had receive a diagnosis of clinical AN or BN with a mild to moderate severity (according to DSM-5 criteria) at the beginning of the *e*LoriCorps Program, (b) be between 12 and 30 years old at the moment of the evaluations and (c) be a self-identified girl/woman. Patients with self-reported severe comorbid psychiatric conditions (e.g., personality disorders, severe anxiety or depression, psychosis, etc.) were excluded.

### Measures

Body Mass Index (BMI = Kg/m^2^) was collected from weight and height data with a calibrated balance and a stadiometer.

Dimensions of EE were measured with the French version of the Exercise and Eating Disorder questionnaire (Danielsen et al., 2015). This tool is a self-report questionnaire with 21 statements regarding the last four weeks. The main EED questionnaire yield a mean Global Qualitative Score of EE based on 18 items from four subscales: (a) Compulsive exercise (ex., *It feels wrong if I cannot be active every day*), (b) Positive and healthy exercise (ex., *I like to exercise with other people*), (c) Awareness of bodily signals (ex., *I notice when I get tired*), (d) Weight and shape exercise (ex., *I am physically active to burn calories*). The response format is a six-point Likert scale ranging from “Never” (0) to “Always” (Rizk et al., 2015), and positive statements are scored in reverse so higher scores indicate more compulsivity and unhealthy exercises. Scores of the Global Qualitative scale range from 0 to 5. Three additional items used by Danielsen et al. (2015) assessed the quantitative dimensions of EE, with ratings of the amount of exercise in terms of frequency, intensity, and duration. The mean Global Quantitative Score of EE was calculated by averaging the scores of these three additional items once they were rated on the same denominator. Scores of the Global Quantitative scale range from 1 to 4. The internal consistency of this instrument indicates a Cronbach coefficient of 0.96.

Dimensions of physical self-perception were assessed with the French version of the Physical Self Inventory (Maïano et al., 2008). This 12-item instrument measures: (a) Global self-esteem (how people perceive themselves in general; ex., *I have a good opinion of myself*), (b) Physical self-worth (general feelings of happiness, satisfaction, and pride in the physical field; ex., *Globally, I’m proud of what I can do physically*), (c) perceived Sport competence (athletic ability and ability to learn sports; ex., *I do well in sports*), (d) perceived Physical condition (stamina and fitness; ex., *I would be good at aerobic exercise*), (e) perceived Physical attractiveness (how patients perceive their ability to maintain an attractive body over time; ex., *I have a nice body to look at*), and (f) perceived Physical strength (perceived strength and muscle development; ex., *I’m physically stronger than most people*). Answers are rated on a six-point Likert scale of agreement ranging from “Not at all” to “Absolutely” and the scores of the subscales range from 0 to 5. The internal consistency of this instrument indicates Cronbach coefficients between.76 and.90 (depending on the scales).

**Figure 1:**
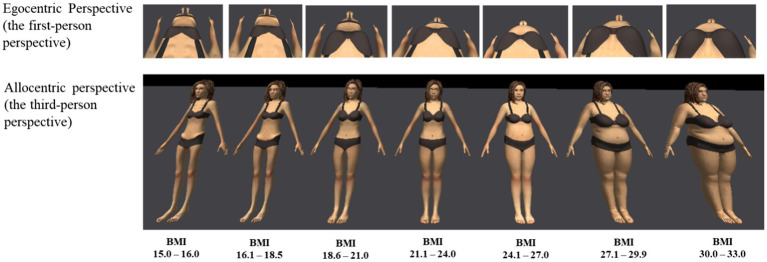
Egocentric and allocentric perspectives of eLoriCorps Immersive Body Rating Scale used with females participants. The screenshots are not perfect replicas of images displayed in VR because of variations in viewpoints in the egocentric illustration and a fisheye distortion in the allocentric illustration. Illustration reproduced from Monthuy-Blanc et al. (2022) with the permission of the authors under the Creative Commons Attribution license.

The *e*LoriCorps Immersive Body Rating Scale 1.1 (*e*LoriCorps-IBRS; Monthuy-Blanc et al., 2022) was used to measure body image dissatisfaction and body image distortion according to allocentric and egocentric perspectives (see Figure 1, note the screenshots are not perfect replicas of images displayed in VR because of variations in viewpoints in the egocentric illustration and a fisheye distortion in the allocentric illustration). The *e*LoriCorps-IBRS requires immersions in VR [see Monthuy-Blanc et al., (Monthuy-Blanc et al., 2020) for a description of the hardware] to examine a series of seven virtual bodies representing a gradient of body size and shape from underweight (VB#1) to overweight (VB#7). In the allocentric perspective, participants walked around and examined each body in the lineup to decide which one represents their own body size, and which one represents the ideal body they want to look like. In the egocentric perspective, participants looked down and experienced being in each of the seven bodies before selecting which one best represents their own body size and the ideal body they want to look like. Scores corresponding to the virtual body selected ranged from 1 to 7 and were transformed into body dissatisfaction and distortion scores. The Allocentric Perspective Body Dissatisfaction score is calculated as the difference between the virtual body viewed by participants in the lineup and selected to represent their own, minus the ideal one selected from the lineup. The Egocentric Perspective Body Dissatisfaction score is calculated as the difference between the virtual body experienced by participants as representing their own, minus the one experienced as their ideal body. A positive score suggests a desire for a thinner body. The Allocentric Perspective Body Distortion score is calculated as the difference between participants’ actual BMI minus the BMI of the virtual body viewed in the lineup and selected to represent their own. The Egocentric Perspective Body Distortion score is calculated as the difference between participants’ actual BMI minus the BMI of the virtual body experienced as representing their own. A positive score suggests the perception of a thinner body than the actual BMI.

To describe the sample, reasons for practicing physical and/or sports activities at least once a week were documented by asking the following: Which physical activity do you practice and for which reason do you practice it? Check one or more of the following choices: well-being, treatment of a physical or psychological problem, performance, or weight loss.

### Ethical considerations

This study was approved by the Université du Québec à Trois-Rivières’ ethics committee before starting the recruitment (CER-16-223-07.23). At the time of admission to the *e*LoriCorps program, participants provided free and informed consent. The aim of the study was explained and participants could ask questions before providing verbal and written consent.

### Statistical analysis

The data was entered into Excel software and exported and analyzed using IBM SPSS Statistics (v.24). The descriptive data was analyzed using mean and standard deviation. The normality of distribution of each variable was checked with Skewness and Kurtosis tests. According to George and Mallery (George and Mallery, 2016), the results indicates that data was normally distributed, with Skewness and Kurtosis statistics situated between −1,169 and 0.796. Pearson’s correlation analyses were used. Because of the exploratory nature of the study, many correlations are reported only to inform readers about the relationships between the instruments and among their subscales. Therefore, no corrections are applied to control for inflation of Type 1 error and statistically significant results will be interpreted with accordingly by focusing mostly on effect sizes, according to Cohen’s guidelines (Cohen, 1988). Several significant correlations pertain to factors from the same questionnaire and therefore will receive less attention than correlations between instruments. Comparisons between correlations mentioned in the research questions that reached statistical significance were performed according to procedures described by Meng et al. (1992).

To explore with semi-partial correlations the effect of statistically significant physical self-perceptions and body image disturbances on EE, multiples regression analyses were planned to be conducted independently for the Global Qualitative Score and the Global Quantitative Score of the EED questionnaire. The significance level was set at *p* < 0.05 to analyze predictors that would reach statistical significance during the main analyses, with a focus on interpreting semi-partial correlations.

## Results

From the initial 44 participants, 8 did not complete the immersion in VR because they arrived in late for their appointment. The final sample consists of 36 girls/women with a diagnosis of AN (*N* = 22, mean age: 20.24 ± 4.3 years; mean BMI: 18.34 ± 2.6 kg/m^2^) or with a diagnosis of BN (*N* = 14, mean age: 23.36 ± 4.6 years; mean BMI: 23.12 ± 3.7 kg/m^2^). Descriptive statistics are reported in Table 1. When providing reasons for exercising at least once a week, 18% of participants reported two different reasons, 62% reported three different reasons, and 20% reported four different reasons.

**Table 1:** Descriptive analyses of the sample (*N* = 36).

	Mean (standard déviation)
Age	21.49 (4.63)
Body mass index (Kg/m^2^)	20.07 (3.60)
Frequency of exercising	
Never	5.6%
Less than once a week	8.3%
Once a week	5.6%
Two to three times a week	30.6%
Every day	50.0%
Reasons for exercising at least once a week	
Physical and psychological well-being	52.0%
Improve performance	12.0%
Treatment of a physical or psychological problem	46.0%
Losing weight	68.0%
Exercise and Eating Disorder Questionnaire	
Compulsive Exercise	2.31 (0.98)
Positive and Healthy Exercise	2.12 (1.18)
Awareness of Bodily Signals	1.47 (0.83)
Weight and Shape Exercises	3.09 (1.46)
Global Qualitative Score	2.22 (0.70)
Global Quantitative Score	2.92 (0.64)
Physical Self Inventory	
Global Self-esteem	2.23 (1.03)
Physical Self-worth	2.82 (1.11)
Perceived Sport Competence	2.90 (1.58)
Perceived Physical Condition	2.77 (1.78)
Perceived Physical Attractiveness	2.76 (1.05)
Perceived Physical Strength	2.14 (1.16)
*e*LoriCorps-Immersive Body Rating Scale	
Allocentric Perspective Body Dissatisfaction	1.00 (1.60)
Egocentric Perspective Body Dissatisfaction	0.94 (1.41)
Allocentric Perspective Body Distortion	1.50 (1.46)
Egocentric Perspective Body Distortion	1.63 (1.52)

The associations between qualitative and quantitative dimensions of EE, dimensions of the physical self, and allocentric/egocentric perspectives of body image in a sample of young adults with ED are reported in Table 2 for all scales and subscales.

**Table 2:** Pearson’s correlations among qualitative and quantitative dimensions of excessive exercises, dimensions of the physical self, and allocentric/egocentric perspectives of body image in a sample of young adults with ED, with correlations of main interest identified by boxes (*N* = 36).

	1	2	3	4	5	6	7	8	9	10	11	12	13	14	15	16
Excessive exercise																
1. Compulsive exercise		−0.16	0.19	0.66^**^	0.85^**^	0.54^**^	−0.09	−0.05	0.29	0.25	−0.15	−0.02	0.34^*^	0.17	0.17	−0.02
2. Positive and healthy exercise			0.21	−0.09	0.23	0.43^**^	−0.30	−0.36^*^	−0.51^**^	−0.50^**^	−0.31	−0.31	0.16	0.38^*^	0.28	0.43^*^
3. Awareness of bodily signals				0.21	0.52^**^	0.09	−0.52^**^	−0.42^*^	−0.17	−0.16	−0.52^**^	−0.35^*^	0.15	0.24	−0.28	−0.07
4. Weight and shape exercises					0.78^**^	0.50^**^	−0.21	−0.19	0.14	0.08	−0.42^*^	−0.20	0.37^*^	0.45^**^	0.09	0.14
5. Global Qualitative Score						0.40^*^	−0.35^*^	−0.32	0.03	0.01	−0.47^**^	−0.26	0.42^**^	0.46^**^	0.16	0.16
6. Global Quantitative Score							−0.06	0.09	0.39^*^	0.39^*^	−0.03	0.09	0.14	0.11	−0.19	−0.18
Perceived physical self																
7. Global self-esteem								0.64^**^	0.28	0.40^*^	0.58^**^	0.29	−0.47^**^	−0.50^**^	−0.10	−0.06
8. Physical self-worth									0.58^**^	0.58^**^	0.58^**^	0.62^**^	−0.44^**^	−0.48^**^	−0.15	−0.04
9. Sport competence										0.88^**^	0.35^*^	0.46^**^	0.0	−0.23	−0.31	−0.35^*^
10. Physical condition											0.42^*^	0.38^*^	−0.1	−0.35^*^	−0.36^*^	−0.37^*^
11. Physical attractiveness												0.42^**^	−0.39^**^	−0.64^**^	−0.11	−0.29
12. Physical strength													−0.14	−0.25	−0.05	−0.15
Allo/egocentric perspectives																
13. Allocentric – Body Dissatisfaction														0.77^**^	0.40^*^	0.10
14. Egocentric – Body Dissatisfaction															0.25	33
15. Allocentric – Body Distortion																0.71^**^
16. Egocentric – Body Distortion																

* *p* < 0.05;

** *p* < 0.01.

Numbers in the first row refers to the variables listed in the first column. Statistically significant correlations are highlighted in bold to facilitate readability.

The results revealed different patterns of size of correlations with dimensions between Quantitative and Qualitative scores of EE and physical self-perceptions scores. The Qualitative scores of EE correlated significantly, inversely and with a medium effect size with perceived Physical attractiveness, and to a smaller extent with Global self-esteem and Physical self-worth. The Quantitative score of EE correlated positively to a significant and medium extent with perceived Sport competence and perceived Physical condition. The differences in correlations between Qualitative versus Quantitative scores were statistically significant for Physical attractiveness and perceived Physical condition (see Table 3). Differences in the size of the correlation patterns between Qualitative and Quantitative scores were observed among subscales. Statistically significant correlations were also found between the Global Qualitative score of EE, and also one of its subscales, with both Allocentric and Egocentric perspectives of body image dissatisfaction. Although correlations between measures of body image were statistically significant for the Qualitative EE score and not significant for the Quantitative score, the direct comparisons of correlations with the Qualitative versus Quantitative scores did not reach statistical significance (see Table 3). Finally, correlations with measures of body image distortion were all very small and not statistically significant, except for Positive and healthy exercises assessed in the Egocentric perspective.

**Table 3:** Comparisons among pairs of correlations (*N* = 36).

Correlations compared	Difference between correlations	*Z*	Exact *p* (bilateral)
Qualitative vs. Quantitative EE Score with Global self-esteem	−0.29	−1.57	0.12
Qualitative vs. Quantitative EE Score with Sport competence	−0.36	−1.94	0.05
Qualitative vs. Quantitative EE Score with Physical condition	−0.38	−2.05	0.04
Qualitative vs. Quantitative EE Score with Physical attractiveness	−0.44	−2.41	0.02
Qualitative vs. Quantitative EE Score with Allocentric – Body Dissatisfaction	0.28	1.55	0.12
Qualitative vs. Quantitative EE Score with Egocentric - Body Dissatisfaction	0.35	1.95	0.05
Allocentric vs. Egocentric Score of Body Dissatisfaction with Global self-esteem	0.03	0.38	0.77
Allocentric vs. Egocentric Score of Body Dissatisfaction with Physical self-worth	0.04	0.39	0.7
Allocentric vs. Egocentric Score of Body Dissatisfaction with Physical condition	0.25	2.18	0.03
Allocentric vs. Egocentric Score of Body Dissatisfaction with Physical attractiveness	0.25	2.55	0.01
Allocentric vs. Egocentric Score of Body Distortion with Sport competence	0.04	0.32	0.75
Allocentric vs. Egocentric Score of Body Distortion with Physical condition	0.01	0.08	0.94

In terms of dimensions of the physical self-perceptions that have not yet been mentioned in the above paragraph, correlations with body image dissatisfaction were often large, negative, and statistically significant. Patterns of correlations for Allocentric and Egocentric perspectives were similar in effect sizes for Global self-esteem and Physical self-worth, and statistically different (see Table 3) in sizes for perceived Physical attractiveness and perceived Physical condition. Body image distortion was negatively correlated, a with medium effect size, with perceived Physical condition according to both perspectives and with perceived Sport competence in the egocentric perspective only. The differences in Allocentric and Egocentric perspectives were mostly noticeable when looking at the differences in size of correlations with Compulsive exercise, Positive and healthy exercise and perceived Sport competence.

Given the shared variance among some of the dimensions of the physical self-perceptions and body image measures, semi-partial correlations were examined with a multiple regression predicting EE. Only the Qualitative score of the EE was studied, because of a problem of multicollinearity (*r* = 0.88) among the only two potential predictors (perceived Sport competence and perceived Physical condition) of the Quantitative score. EE Four measures were included in the regression: Global self-esteem, perceived Physical attractiveness, Allocentric perspective of Body Dissatisfaction and Egocentric perspective of Body Dissatisfaction. To control for multicollinearity among the Allocentric and Egocentric perspectives of Body Dissatisfaction, the analysis was also performed using only the measure from the Egocentric perspective, and results were similar to those reported in this article.

The regression model was statically significant [*F*(4,34) = 3.88, *p* = 0.012, Adj *R*^2^ = 0.25], with a 34.% of variance explained in Global Qualitative EE score. As shown by the semi-partial (sr) correlations in Table 4, when controlling for shared variance, only perceived Physical attractiveness was statistically significantly related to the Qualitative dimensions of EE.

**Table 4:** Summary of multiple regression analyses for variables predicting the Global Qualitative dimensions score of excessive exercise.

Predictor entered in the model	*B*	SE B	*β*	*p=*	*part.*	*sr*
Global self-esteem	0.083	0.13	0.13	0.51	0.12	0.1
Perceived Physical attractiveness	−0.329	0.14	−0.55	0.025	−0.39	−0.35
Allocentric perspective Body Dissatisfaction	0.07	0.1	0.17	0.49	0.13	0.1
Egocentric perspective Body Dissatisfaction	0.028	0.13	0.06	0.83	0.04	0.03

## Discussion

### General

The sample consist of young adults with AN or BN with an average BMI higher than in many studies of ED. The clinical program in which the patients were recruited addresses clinical cases of “mild” to “moderate” severity (according to DSM-5 criteria), which may explain a higher BMI than in other clinical samples. They reported exercising for several reasons, but mostly to lose body weight. This study aims to document the association between the various dimensions of EE – both in terms of how people engage in these exercises (qualitative dimensions) and with which quantity (quantitative dimensions) – with six dimensions of the physical self-perceptions as well as body dissatisfaction and body distortion image assessed from the allocentric and the egocentric perspectives.

Our main results support the observation by Danielsen et al. (2015) that the qualitative dimension of EE score was mostly correlated with compulsive exercise and weight and shape exercises. Global self-esteem and body dissatisfaction were positively associated with the qualitative dimension of EE, as seen in previous correlative studies (Brewerton et al., 1995; Solenberger, 2001). However, our results revealed statistically significant differences in the pattern of correlations with Perceived physical self when EE was measured with Global Qualitative versus Quantitative scores. When examining results between EE and the perceived physical self, the qualitative dimension of EE was correlated mainly and inversely with the perceived physical attractiveness. This was not captured by the quantitative assessment of EE. This relationship remained statistically significant after controlling, with a multiple regression, for global self-esteem and body dissatisfaction. This is consistent with prior knowledge about the role of perceived physical appearance in AN and BN and body dissatisfaction (Bruch, 1962; Crocker and Wolfe, 2001; Fairburn et al., 2003). The difference in information revealed by qualitative versus quantitative dimensions of EE was also revealed in the pattern of correlations with perceived Physical condition. These results support the importance of considering EE and self-esteem as multidimensional constructs. Qualitative and quantitative dimensions of EE provide different information relevant in the assessment of ED. Perceived physical self is also multidimensional, providing different information for the assessment of ED. It may explain some inconsistencies among studies that used unidimensional approaches (Davis et al., 1999; Monthuy-Blanc et al., 2012; Levallius et al., 2017). But globally, these results are coherent with a large meta-analysis of 106 studies conducted in the general population that demonstrated that exercise is positively related to global self-esteem.

Our results also suggest that the feeling to be competent in sport and to be able to stay in shape may motivate clinical population with ED, particularly in anorexia nervosa and bulimia nervosa, to engage in EE more often, as suggested by others (Probst et al., 2014). Because the sample of the present study is composed by people with low to moderate ED severity, it is possible that global self-esteem is less influenced by specific physical self-perceptions than in patients with ED with severe to extreme severity. Our results are consistent with Martin et al.’s (Martin et al., 2016) who found that sport competence and physical condition were the only two significant contributors to the variance of amount of exercise in general population. According to the Monthuy-Blanc et al.’s study (Monthuy-Blanc et al., 2012) on the directionality of the relationships between global self-esteem and physical self dimensions in outpatients’ girls with anorexia, global self-esteem of severe patients may be directly influenced by physical self-perceptions. In-depth idiographic analysis suggested a bi-directional determinism, with reciprocal and simultaneous bottom-up and top-down relations (Feist et al., 1995; Marsh and Yeung, 1998). The significant correlation observed between the quantitative dimensions of EE, compulsive exercise, positive and healthy exercise, and weight and shape concerns, are worth noting. They are consistent with the likely unhealthy contribution of the intensity of the EE. The measure of EE used in this study, the Exercises and Eating Disorders Questionnaires (EEDQ; 43), provided a nuanced assessment of four clinical constructs assessing the qualitative dimension of EE.

The contrasting results between the allocentric and the egocentric perception of body dissatisfaction obtained when examining perceived Physical condition and physical attractiveness is also a significant contribution of the current study. Both perspectives correlated significantly with the qualitative score of EE, with Global self-esteem and with Physical self-worth. But assessing body dissatisfaction from the egocentric perspectives reveals unique perspectives on the significant relationship with Physical attractiveness and perceived Physical condition. This difference in perspective may be explained by the egocentric perspective reflecting perceptual-affective construction of body image deriving mainly from intra-individual comparisons (Monthuy-Blanc et al., 2020). This is in contrast with the allocentric perspective reflecting the cognitive-affective construction of body image depending to inter-individual comparisons (Monthuy-Blanc et al., 2020). Also, only the allocentric perspective correlated significantly with compulsive exercise, while only the egocentric perspective correlated significantly with Positive healthy exercise. In sum, it is interesting to assess both perspectives when interpreting the functions of EE in ED.

Results are consistent with recent studies in adult and adolescent community of sample (Corno et al., 2018; Monthuy-Blanc et al., 2020) documenting the role of the egocentric perspective as a perceptual-sensitive-affective construction of the body. EE may involve the desire to change body shape without paying attention to body signals. Consequently, for clinicians interested in understanding perceived physical self and EE, looking at perceived physical attractiveness and weigh and shape concerns will be more revealing than how compulsively the exercises are done. This is different from examining EE to distinguish between people with and without ED, where compulsive exercises, followed by weight and shape concerns, are more revealing according to Danielsen et al. (2015). In sum, for people with ED, quantitative assessment of EE seems to be related to the perception of what their body can do, in terms of performance, and the qualitative dimensions seems to be related to what they body can look like, in terms of weight and shape.

Results show that the awareness of bodily signals during exercise is inversely correlated with qualitative dimensions of EE. This suggests that the less the participant were able to identify the signals of fatigue and pain, the more they were inclined to continue their exercise. Inversely, it is possible that the increase in quantity of exercise could be related to participants being less able to identity and listen their bodily signals. A parallel can be drawn between these results and behavioral addiction in which the benefits associated with continuing to “consume” exercise are greater for the individual than stopping to exercise. A recent large-scale study found that AN and intense exercises share the same genetic predisposition (Watson et al., 2019). EE would bring about the same neurological pleasure response as that generated by fasting in ED. Our findings on this topic deserve to be further studied and interpreted in the context of a continuum from occupation-wellness to occupation-symptom (Ouellet, 2020; St-Pierre et al., 2022).

### Strengths and limitations

There were several strengths to this study, and an important one is the assessment of both qualitative and quantitative dimensions of EE. It avoids the shortcomings of categorizing EE according to inconsistent definitions. Additionally, key physical self-perception dimensions were included to ascertain which dimensions were best predictors of EE. A multiple regression analysis was necessary to ascertain which measures were independent predictors of EE while accounting for shared variance. The use of psychometric tools that consider allocentric and egocentric perspectives provides a more nuanced picture about body dissatisfaction and body distortion. Finally, the use of a clinical sample of non-hospitalized patients militates in favor of the generalizability of the results.

A limitation of this study was the exclusively female sample of patients. This precludes generalizability to males with an eating disorder. Self-perceptions could also differ among participants from different age groups or those with AN versus BN. The study did not have the statistical power to control for these variables in the main analyses or the regression. Also, we used retrospective data when assessing EE quantitative and qualitative scores that may be related to recall biases. The sample size and number of correlations conducted without controlling for Type 1 error call for caution when interpreting correlations coefficients. Consequently, the results of this exploratory study should be interpreted with caution and replication is warranted.

### Clinical implications

This study shed light important clinical implications. Among other, the results of this study highlight the importance of nuances when looking at EE. Results suggest that quantity of exercise is not necessarily pathological. This is how the exercise is done (i.e., the qualitative dimensions) that may be associated with ED. The compulsive nature of EE may help differentiate people with and without ED (Danielsen et al., 2015), but compulsiveness may be mostly related to body dissatisfaction assed from the traditional perspective and exercising to change body shape without paying attention to body signals may be more revealing of the nature of the potential functional or dysfunctional effect of exercise on the course of the eating disorder.

The results of this study suggest that exercise in exerciser patients is not necessarily a problem but should be part of the solution for recovery. Indeed, patients who perceived themselves as competent in physical abilities domains may benefit from a treatment in which exercise is considered an important sphere of identity for the person. Since the control of eating as well as body shape and weight become the overinvested identity domain in eating disorder patients, investing other areas that are important for the patient and that are related to a high perception of competence may help to lead to a sense of valorization in an area other than physical appearance. Nevertheless, it has been shown that exerciser patients consider exercise as healthier than other behaviors of weight control (Johnston et al., 2011). Thus, to avoid pathological over-investment in exercise, working on modifying reasons for exercising must be done throughout the treatment. This may help to go from the occupation-symptom to the occupation-well-being. Thus, adapted physical activity becomes a key to allow exerciser patients to give a more functional meaning to exercise.

Indeed, the inclusion of adapted physical activity in eating disorder exerciser or non-exerciser patients is related to a decrease of anxiety and depressive symptoms (Noetel et al., 2017), EE attitudes and behaviors (Calogero and Pedrotty, 2004), drive for thinness and body dissatisfaction (Thien et al., 2000; Cook and Hausenblas, 2008). To reconnect patients with ED with their bodily sensations and decrease motivations based on physical appearance control, caregivers should help patients change the focus on the “what my body looks like” to “what my body can do” that remains healthy.

## Conclusion

In sum, this study shows that it is necessary to assess all dimensions of perceived physical self in relation to quantitative and qualitative dimensions of EE. It could be worth distinguishing exercise as a functional behavior motivated by positive perceived physical self leading to well-being, and EE as a symptom driven by disturbances of perceived physical self. Overall, the results indicate that the EE quantitative and qualitative dimensions provide complementary information necessary for a holistic understanding of exerciser patients. A continuum from wellness-occupation to symptom-occupation appears to be a relevant conceptual representation leading to the necessity to conduct curvilinear analysis. Importantly, this conception highlights the importance of crossing perspectives between disciplines in order to better treat this phenomenon. In particular, the results of this paper invite to question the place of exercise in the ED programs because of the functional valence that exercise can translate when the conduct is guided by transdisciplinary clinical teams.

## Data availability statement

The raw data supporting the conclusions of this article will be made available by the authors, without undue reservation.

## Ethics statement

This study was approved by the Université du Québec à Trois-Rivières (Quebec, Canada) ethics committee before starting the recruitment (CER-16-223-07.23). The patients/participants provided their written informed consent to participate in this study.

## Author contributions

MO and JM-B: conceptualization, data collection, and project administration. MO, JM-B, SB, and MR: methodology. JM-B: software and funding acquisition. MO, JM-B, and SB: formal analysis. MO, JM-B, and RP: writing—original draft preparation. JM-B and SB: supervision. MO, JM-B, RP, MR, and SB: writing—review and editing. All authors contributed to the article and approved the submitted version.

## Funding

This research was funded by the foundation of Université du Québec à Trois-Rivières (from Foundations of RBC Royal Bank and Lemaire family), the Social Sciences and Humanities Research Council of Canada (CRSH-SAVOIR-430-2018-00970-PIE-IC), and partly by the Canada Research Chairs programs (Canada Research Chair in clinical cyberpsychology # 950–231039).

## Conflict of interest

SB is president of, and own shares in, Clinic and Development In Virtuo, a company that distributes virtual environments, and conflict of interest are managed under UQO’s conflict of interest policy.

The remaining authors declare that the research was conducted in the absence of any commercial or financial relationships that could be construed as a potential conflict of interest.

## Publisher’s note

All claims expressed in this article are solely those of the authors and do not necessarily represent those of their affiliated organizations, or those of the publisher, the editors and the reviewers. Any product that may be evaluated in this article, or claim that may be made by its manufacturer, is not guaranteed or endorsed by the publisher.

## Footnotes

^1^ uqtr.ca/Loricorps
